# Reconstruction-Assisted Band Selection for Non-Destructive Prediction of Citrus Soluble Solids Content from VNIR Hyperspectral Images

**DOI:** 10.3390/foods15101774

**Published:** 2026-05-18

**Authors:** Junjie Zhao, Siya Liu, Fengyong Yang, Long Cheng, Fang Hu, Sixing Xu, Lei Shan

**Affiliations:** 1 School of Physics and Electronics, Hunan University, Changsha 410082, China; jjzgao@hnu.edu.cn (J.Z.); siyaliu@hnu.edu.cn (S.L.); lcheng@hnu.edu.cn (L.C.); 2 College of Semiconductors (College of Integrated Circuits), Hunan University, Changsha 410082, China; yangfengyong@hnu.edu.cn; 3 School of Artificial Intelligence and Robotics, Hunan University, Changsha 410082, China; hufang@hnu.edu.cn

**Keywords:** hyperspectral imaging, band selection, reconstruction, soluble solids content, deep learning, interpretability

## Abstract

The increasing demand for better fruit flavor and eating quality has driven the need for rapid and non-destructive assessment of internal attributes to support fruit grading and precision supply. Visible–near-infrared hyperspectral imaging (VNIR-HSI) provides rich spectral–spatial information for evaluating sweetness in citrus fruit, but its practical use is constrained by high spectral dimensionality, redundancy, and system cost. Here, we propose a reconstruction-assisted, attention-guided band-selection framework for non-destructive prediction of soluble solids content (SSC) in Shimen honey mandarins. The framework integrates spectral–spatial attention, probability-based differentiable band selection, and full-band reconstruction into a unified end-to-end architecture, enabling compact and informative band learning. Using 952 samples, the model selected 56 informative bands from the original 176-band hyperspectral data and achieved competitive SSC prediction on the test set (RMSE = 0.63 °Brix, R^2^ = 0.80) while maintaining high-fidelity reconstruction of the full-band hyperspectral cube from the compact input (peak signal-to-noise ratio, PSNR = 36.47 dB; structural similarity index, SSIM = 0.89). These findings support the proposed framework as a methodological proof of concept for non-destructive citrus quality evaluation, indicating that substantial spectral compression can be achieved under the current VNIR setting while largely preserving predictive performance. The selected bands may provide candidate spectral regions for future compact citrus-quality sensing systems.

## 1. Introduction

Achieving better fruit flavor and eating quality remains an important objective in modern citrus production and marketing, creating demand for rapid and non-destructive evaluation of internal quality attributes for grading and precision supply. Among these attributes, soluble solids content (SSC, commonly expressed as °Brix) is one of the most widely used indicators related to perceived sweetness and overall eating quality in citrus fruit. Although SSC can be measured rapidly and inexpensively in expressed juice using refractometers, such measurements are destructive and therefore unsuitable for high-throughput screening of intact fruit. This limitation has motivated sustained interest in optical sensing methods for non-destructive evaluation of citrus internal quality [1,2,3,4,5,6].

Near-infrared (NIR) spectroscopy has shown practical value for SSC determination, but conventional point-based measurements sample only a limited local region and may not adequately represent intact citrus fruit, whose optical response is influenced by peel structure, waxing treatment, scattering behavior, and internal compositional heterogeneity [5,6,7,8,9,10]. Visible–near-infrared hyperspectral imaging (VNIR-HSI) provides a more informative alternative by combining spectroscopy with imaging and preserving both spectral absorption features and their spatial distribution within a three-dimensional data cube. Compared with point spectroscopy, HSI is therefore better suited to intact-fruit SSC evaluation because it can capture not only chemical information related to sweetness, but also spatial variation across the fruit surface [2,3,4,11,12,13,14,15].

Despite these advantages, the practical deployment of hyperspectral systems remains constrained by the high dimensionality and redundancy of full-band data [2,3,4,14,15]. Adjacent wavelengths are often strongly collinear, which increases acquisition cost, storage burden, and computational latency, while also aggravating overfitting in relatively small-sample regression tasks. Consequently, spectral compression is not merely a modeling convenience, but a necessary step toward translating laboratory HSI into cost-effective multispectral sensing systems. In many previous citrus SSC studies, wavelength selection and regression have been treated separately [16,17,18,19,20,21]. Typical two-stage pipelines first screen informative wavelengths using heuristic or statistical methods such as competitive adaptive reweighted sampling (CARS), and then fit an independent regression model, often PLSR or another shallow learner. These strategies are simple and interpretable, which explains their continued popularity in practical SSC studies. However, because the selected subset is not optimized directly for the final SSC objective, it may be sensitive to sampling variation, scattering noise, and batch effects, and may fail to preserve the most task-relevant information for downstream prediction [21,22,23,24,25]. In addition, models built only on selected spectra usually make limited use of the spatial structure available in hyperspectral images.

Recent advances in deep learning have improved representation learning from hyperspectral or image-like inputs [16,17,18]. Full-band CNN- and Transformer-based regressors can learn end-to-end mappings from the complete spectral cube and provide strong predictive baselines for fruit quality evaluation. However, they still rely on the full hyperspectral input at inference time and therefore do not solve the engineering problem of spectral compression. In parallel, deep band-selection frameworks such as BS-Net and DARecNet have moved toward data-driven compact representation learning by coupling band weighting with reconstruction [26,27,28,29]. Compared with handcrafted wavelength screening, these approaches provide a stronger basis for adaptive band learning. Nevertheless, reconstruction-oriented or unsupervised selection may favor bands that are useful for restoring the overall input distribution rather than those most sensitive to the downstream SSC target. This distinction is particularly important for intact citrus fruit, where SSC is inferred indirectly through coupled changes in pigments, maturity status, water-related absorption, tissue structure, and surface optical properties rather than through a single isolated spectral feature [11,12,13,20]. Therefore, an effective compact-band strategy for citrus SSC sensing should remain directly guided by the prediction task while still preserving sufficient spectral–spatial information to avoid overly narrow, dataset-specific selection.

To address these issues, this study proposes a reconstruction-assisted, attention-guided band-selection framework for non-destructive SSC prediction in Shimen honey mandarins. The framework is designed to integrate spectral–spatial attention, differentiable compact-band learning, full-band reconstruction, and SSC regression within a unified select–reconstruct–predict pipeline. Rather than treating wavelength selection, reconstruction, and prediction as independent steps, the proposed strategy aims to learn a compact spectral representation that is simultaneously constrained by downstream SSC prediction and by the preservation of broader hyperspectral information.

The central hypothesis of this study is that compact bands selected under both prediction-oriented supervision and reconstruction-assisted regularization can retain more task-relevant spectral–spatial information than bands selected only by heuristic screening or reconstruction-oriented criteria. Accordingly, the objectives of this work were (i) to develop an end-to-end framework for task-driven compact-band learning from VNIR hyperspectral images of intact citrus fruit; (ii) to evaluate whether substantial spectral compression can be achieved while maintaining useful SSC-prediction capability; and (iii) to examine whether the learned wavelength subset and the associated trade-off between compactness, reconstruction fidelity, and prediction performance can provide practical guidance for future compact multispectral citrus-quality sensing.

## 2. Materials and Methods

### 2.1. Fruit Samples and SSC Measurement

**Figure 1:**
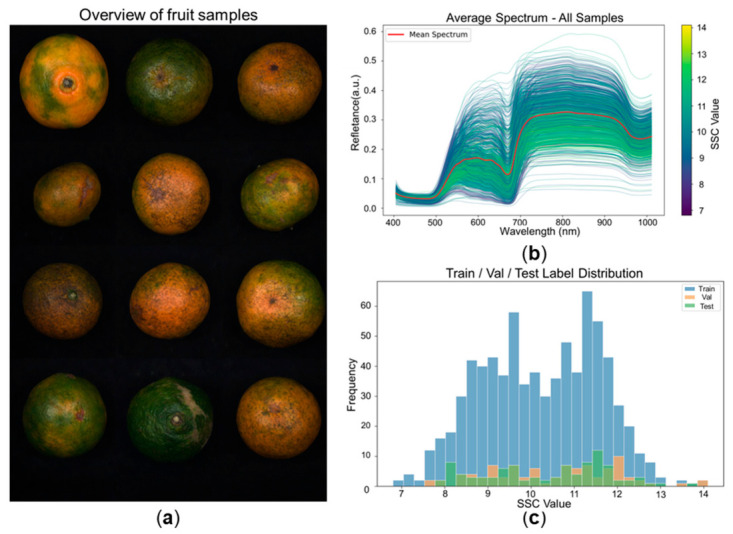
Overview of fruit samples, spectral characteristics, and dataset split. (**a**) Representative waxed Shimen honey mandarins used in this study, showing natural variation in peel color, surface texture, and external appearance. (**b**) Average reflectance spectra of all samples in the VNIR range; the line color corresponds to the SSC label of each fruit, illustrating both the shared global spectral shape and the inter-sample variability associated with internal quality differences. (**c**) Histograms of SSC labels for the training, validation, and test subsets after distribution-friendly splitting, showing that the three subsets follow comparable label distributions.

Shimen honey mandarins were used in this study. All fruit samples were purchased from local retail channels and were labeled as originating from Shimen. This commercial-source sampling was intended to reflect practical postharvest circulation and grading conditions. Compared with highly uniform laboratory or orchard-controlled samples, this fruit retained greater natural variation in peel color, surface texture, fruit size, and maturity status, as illustrated in Figure 1a. Such variability provides a challenging and application-relevant scenario for evaluating non-destructive SSC prediction in intact citrus fruit under practical conditions [1,5,7,11,12,13]. The final dataset comprised 952 fruit samples, each paired with one hyperspectral cube and one SSC label.

SSC was used as the physicochemical reference trait in this study. It should be noted that the SSC values were not artificially prepared concentration levels or simulated reference values, but destructively measured values obtained from the actual juice of each fruit. After VNIR hyperspectral image acquisition of each intact fruit, juice was extracted from the same fruit and analyzed using a portable Brix/acid meter (PAL-BX|ACID1, ATAGO Co., Ltd., Tokyo, Japan) [30,31,32]. Three repeated measurements were performed for each sample at room temperature, and the valid readings were averaged to obtain the final SSC value in °Brix. These destructively measured SSC values were used as the physicochemical reference values for model training, validation, and final testing.

### 2.2. VNIR Hyperspectral Imaging System and Spectral Acquisition

Hyperspectral images were acquired using a visible–near-infrared (VNIR) pushbroom hyperspectral imaging system (GaiaField-Pro-V10, Zolix Instruments Co., Ltd., Beijing, China). Illumination was provided by four halogen lamps (50 W each; total power 200 W; DC 12 V). The exposure time was set to 12.4 ms, and the samples were scanned horizontally with a push distance of 20 cm per scan. For radiometric calibration, a standard white reference plate made of polytetrafluoroethylene (PTFE) was used (300 mm × 25 mm × 10 mm; nominal spectral range 350–2500 nm). These settings provided stable VNIR hyperspectral acquisition suitable for intact fruit analysis [2,3,4].

The hyperspectral camera recorded 176 spectral bands spanning 404.7–1010.8 nm. Let the calibrated hyperspectral cube of one fruit be denoted as $X\in R^{\left\{C\times H\times W\right\}}$, where C=176 is the number of spectral bands and H and W represent the spatial dimensions. This spectral range captures both visible-region responses related to peel pigments and near-infrared responses related to water and tissue structure, making it suitable for nondestructive modeling of intact citrus quality [2,3,4,13,20].

Figure 1b shows the average reflectance spectra of all samples, where the line color encodes the corresponding SSC value. Although the curves exhibit a broadly similar overall shape, noticeable inter-sample variation can still be observed across the visible and near-infrared regions. This indicates that SSC-related information is not concentrated in a single isolated wavelength, but is embedded in a complex, overlapping spectral response pattern coupled with spatial heterogeneity.

### 2.3. Image Preprocessing and Dataset Split

The raw hyperspectral intensity values were first converted into relative reflectance using white-reference and dark-reference calibration. The calibrated reflectance R(λ) at the wavelength λ was computed as:

(1) $$R\left(\lambda \right)=\frac{I\left(\lambda \right)-D(\lambda )}{W\left(\lambda \right)-D\left(\lambda \right)}$$

where I(λ) is the raw sensor intensity, W(λ) is the white-reference response, and D(λ) is the dark-reference response. This calibration removes the influence of sensor dark current and illumination nonuniformity, allowing the reflectance of different samples to be compared on a common physical scale [2,3,4].

After calibration, the hyperspectral cubes were read from TIFF files and converted to a unified channel-first format (C, H, W). In the implementation, orientation was resolved using the expected band number whenever possible so that both (C, H, W) and (H, W, C) inputs could be handled robustly. If necessary, the cube was truncated or edge-padded to the target band number. Reflectance values were then normalized to [0,1] by min–max scaling; in the main experiments, percentile-based scaling was used for data loading and split statistics, with the default lower and upper clipping bounds set to the 1st and 99th percentiles, respectively.

For network input, a square patch centered on the fruit was extracted from each cube. In the implementation, the default patch size was 448 × 448 pixels. During training, random cropping was applied within the valid spatial range; if the original image was smaller than the target patch, reflective padding was used first. Online augmentation further included random horizontal flipping, vertical flipping, and 90-degree rotation. During validation and testing, center cropping without augmentation was adopted. This design preserves the main fruit region while reducing irrelevant background variation, which is consistent with the goal of spatial redundancy reduction in intact citrus hyperspectral images [25].

The dataset was split into training, validation, and test subsets at a ratio of 8:1:1. Rather than using naïve random partitioning, a distribution-friendly balanced split strategy was adopted. Specifically, the split considered not only SSC labels, but also global intensity statistics and average spectral profiles. In the implementation, all labeled TIFF files were first filtered by valid sample ID, after which each sample was characterized by its SSC label, mean intensity, and mean spectrum. Samples were then grouped by quantile bins of label and intensity, and an initial assignment was refined by minimizing a split cost that combined histogram divergence, mean/variance alignment, and deviation of subset-average spectra from the global spectral mean. This design was intended to reduce distribution mismatch among subsets and thereby avoid spurious instability in compact-band learning that might otherwise arise from unbalanced sampling. The resulting train/validation/test subsets contained 765, 93, and 94 samples, respectively, and showed comparable summary statistics, as reported in Table 1 and Figure 1c.

### 2.4. Reconstruction-Assisted Task-Driven Framework for SSC Prediction

#### 2.4.1. Core Methodological Idea and Overall Workflow

**Figure 2:**
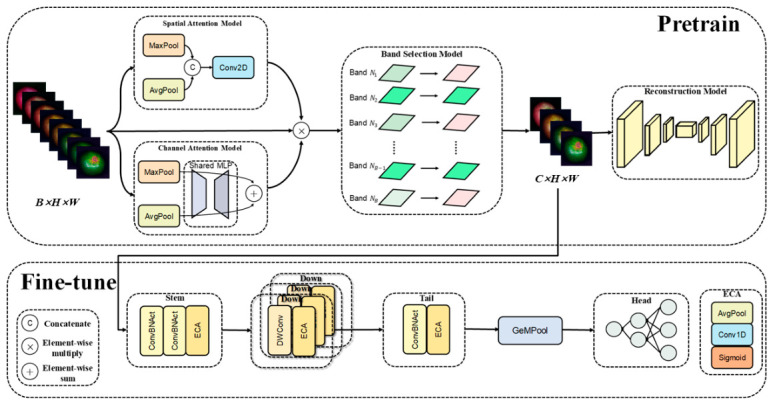
Architecture of the proposed reconstruction-assisted task-driven framework for SSC prediction. The input full-band hyperspectral cube is first processed by the spectral–spatial attention module and the probability-guided compact band learning module to produce a compact K-band representation. The selected representation is then used in two coupled branches: a lightweight U-Net-like reconstruction branch that restores the full-band approximation, and a compact regression branch composed of depthwise separable convolution, ECA, GeM pooling, and a prediction head for SSC estimation. The overall framework follows a unified select–reconstruct–predict paradigm.

The proposed framework follows a unified select–reconstruct–predict paradigm, as illustrated in Figure 2. Given an input hyperspectral cube X, the network first extracts spectral–spatial importance cues through an attention module. A differentiable band-selection module then transforms these cues into band-selection probabilities and selects K number of informative wavelengths to form a compact-band representation $X_{K}$. The selected representation is fed into two coupled branches: a reconstruction branch that restores a full-band approximation $\hat{X}$, and a regression branch that predicts the SSC value $\hat{y}$. Through joint learning, the selected bands are constrained to be not only reconstructive but also directly useful for SSC prediction.

This design addresses a key limitation of conventional two-stage pipelines. A subset chosen only for reconstruction may preserve spectral information well but may not be optimal for the downstream regression target, whereas a subset chosen only for prediction may become unstable if it ignores the global spectral–spatial structure. The present framework, therefore, balances physical fidelity and task relevance by jointly optimizing both branches. Architecturally, it contains four major components: a spectral–spatial attention module, a compact band-learning module based on a probability-based differentiable relaxation, a lightweight U-Net-like reconstruction module, and a compact regression head.

#### 2.4.2. Spectral–Spatial Attention for Informative Feature Enhancement

The attention module is designed to enhance informative responses along both the spatial and spectral dimensions before band selection. In the spatial branch, average pooling and max pooling are first applied along the spectral dimension, and the pooled maps are concatenated and processed by a lightweight convolution to generate a spatial attention mask:

(2) $$M_{S}=\sigma (f_{S}([{Avg}_{C}(X);\;{Max}_{C}(X)]))$$

where [⋅;⋅] denotes concatenation, $f_{S}$ denotes the spatial-attention subnetwork, and σ(⋅) is the sigmoid activation. This branch is conceptually related to the spatial-refinement idea used in CBAM [27], and it highlights spatial regions that are more likely to carry SSC-related reflectance variation while suppressing weakly informative areas such as background or low-response zones.

In the spectral branch, the model estimates the relative importance of each wavelength by compressing the spatial dimensions and learning a band-wise response vector [26,27]:

(3) $$a=\sigma (MLP({GAP}_{\left\{h,w\right\}}(X)))$$

where GAP denotes global average pooling over the spatial dimensions and MLP denotes a lightweight multilayer perceptron. This branch captures inter-band dependency and provides a task-adaptive importance score for each wavelength. By combining the spatial mask $M_{S}$ and spectral attention vector a, the module yields a refined spectral–spatial representation that guides the subsequent differentiable band-selection stage toward more informative regions and bands. Compared with using spectral attention alone, this design is better aligned with intact-fruit hyperspectral data, in which SSC-related information is coupled with both wavelength-dependent reflectance and heterogeneous spatial structure.

#### 2.4.3. Compact Band Learning via a Probability-Based Differentiable Relaxation

The spectral–spatial attention module described in Section 2.4.2 produces a band-importance vector $a=[a_{1},\ldots ,a_{C}]$, which reflects the relative contribution of each wavelength to the current representation. The objective of this subsection is to convert this continuous importance response into a compact representation while preserving end-to-end differentiability. A direct top-K operation would yield a discrete band subset, but its non-differentiability would block gradient propagation from both the reconstruction and regression branches. Therefore, we adopt a probability-based differentiable relaxation, in which compact band learning is decomposed into two coupled steps: estimating a continuous retention preference for each band, and constraining the effective number of retained bands toward a predefined target number of selected bands. This design allows band selection to be optimized jointly with full-band reconstruction and SSC prediction within the unified select–reconstruct–predict framework.

Let $a_{i}$ denote the importance score of the i-th band produced by the spectral attention branch. The corresponding selection probability is defined as

(4) $$p_{i}=\frac{\sigma \left(\left(a_{i}-\tau \right)/T\right)}{\sum_{j=1}^{C}\sigma \left(\left(a_{j}-\tau \right)/T\right)}$$

where τ is an adaptive threshold and T is a temperature parameter. Unlike plain softmax normalization, the thresholded sigmoid term preserves the explicit effect of τ on the selection distribution instead of cancelling it through shift invariance. As a result, bands with responses below the threshold are suppressed, whereas bands with stronger attention responses are assigned higher retention probability. The temperature T further controls the sharpness of this distribution: a larger value encourages exploration in early training, whereas a smaller value produces a more concentrated preference over candidate bands.

Because $p_{i}$ only describes the relative preference among wavelengths and does not by itself guarantee a fixed band budget, an additional compactness constraint is required. Instead of counting hard selected indices, we introduce a smooth pass-count surrogate to estimate the effective number of retained bands:

(5) $$\tilde{K}=\sum_{i=1}^{C}\sigma \left(\frac{a_{i}-\tau }{T_{pass}}\right)$$

where $T_{pass}$ is a smoothing temperature controlling the softness of the counting approximation. The $\tilde{K}$ can be interpreted as a differentiable estimate of how many bands effectively pass the threshold under a soft gate. Based on this surrogate, the compactness regularizer is defined as

(6) $$L_{pass}=\frac{1}{2}{\left(\tilde{K}-K\right)}^{2}$$

where K is the target number of selected bands. This term serves as the compactness component of the overall objective described in Section 2.5. In combination, $p_{i}$ determines which bands are preferentially retained, whereas $L_{pass}$ keeps the effective band count close to the desired compactness level.

During training, the selector is first maintained in a soft form so that gradients from both the reconstruction branch and the SSC regression branch can be propagated through the band-learning module. As training progresses, the selection process becomes increasingly stable, and the learned retention preference is converted into a deployable discrete subset. Specifically, band-selection frequencies are accumulated on the training and validation subsets, and the top-K most consistently retained wavelengths are fixed to construct the final compact representation $X_{K}$. The detailed stage schedule is given in Section 2.5. This progressive transition from soft selection to fixed top-K deployment links differentiable representation learning with the practical requirement of compact spectral design.

Compared with conventional two-stage methods such as CARS-PLSR, the proposed selector is not an offline wavelength screener applied before regression. It also differs from reconstruction-only compact representation learning, in which band choice is driven mainly by input restoration. In the present framework, band learning is directly supervised by the downstream SSC objective while being regularized by reconstruction-assisted information preservation. Therefore, the resulting subset is intended to be not only compact, but also predictive of SSC and sufficiently informative to support full-band reconstruction [13,28,29].

#### 2.4.4. Spectral–Spatial Reconstruction Regularization

A purely task-driven compressed representation may improve regression accuracy, but it can also become overly narrow and overly dependent on a small number of dataset-specific wavelengths, thereby reducing generalizability. To mitigate this risk, a reconstruction-assisted branch is introduced as an auxiliary constraint rather than as an independent end goal. Given the selected compact-band representation $X_{K}$, the reconstruction module outputs a full-band approximation $\hat{X}$ according to

(7) $$\hat{X}=f_{rec}\left(X_{K};\theta_{rec}\right)$$

where $f_{rec}$ denotes the reconstruction network and $\theta_{rec}$ its trainable parameters. In this study, $f_{rec}$ is implemented as a lightweight U-Net-like encoder–decoder with skip connections, which allows the network to combine multiscale context and local detail during reconstruction [33]. A sigmoid activation is used at the output so that the reconstructed reflectance remains in the normalized range.

The role of this branch is to prevent the compact representation from collapsing toward an overly task-specific solution. If the selected subset is too sparse or too narrow, reconstruction quality will deteriorate substantially; conversely, if the retained wavelengths still preserve essential spectral–spatial information, $\hat{X}$ can approximate the original cube X reasonably well. Therefore, the reconstruction branch functions as an auxiliary constraint on information preservation, which makes the proposed framework different from reconstruction-only band-learning methods: here, reconstruction supports task-driven compression, but does not dominate it [28,29,33].

#### 2.4.5. Lightweight Regression Head for SSC Estimation

The regression branch predicts SSC directly from the selected compact-band representation. Let the regression mapping be denoted as $f_{reg}$; the predicted SSC is written as

(8) $$\hat{y}=f_{reg}\left(X_{K};\theta_{reg}\right)$$

where $\theta_{reg}$ denotes the parameters of the regression head. In the proposed framework, this head is implemented as a lightweight convolutional predictor composed of depthwise separable convolution [34], efficient channel attention (ECA) [35], and generalized-mean (GeM) pooling [36]. Depthwise separable convolution improves parameter efficiency by decoupling spatial filtering and channel mixing, ECA adaptively recalibrates feature channels through local cross-channel interaction, and GeM provides a trainable global aggregation mechanism between average pooling and max pooling.

This design is particularly suitable for the compact-band setting. Because the spectral dimension has already been reduced before regression, the predictor should remain sufficiently expressive while avoiding the heavy redundancy of full-band backbones such as ResNet or ViT. More importantly, the regression error is backpropagated through the differentiable band-selection module, which means that the final SSC objective directly affects which wavelengths are retained by the model. This is the essential reason why the proposed framework differs from conventional “select first, regress later” pipelines and why the selected bands can be regarded as task-driven rather than merely reconstruction-optimal.

### 2.5. Progressive Optimization Strategy

Because compact band learning, spectral–spatial reconstruction, and SSC regression are optimized jointly, training stability is critical. In early training, compact band learning should remain exploratory so that informative wavelengths can emerge under the joint influence of reconstruction and prediction. As training proceeds, the compact representation should become more stable and the optimization emphasis should gradually shift toward the SSC prediction objective. Therefore, a progressive optimization strategy was adopted instead of a fixed training scheme. The overall objective is written as

(9) $$L=\alpha \left(t\right)\;L_{rec}+\beta \left(t\right)L_{reg}+\lambda \;L_{pass}$$

where $L_{rec}$ is the reconstruction loss (mean squared error between $\hat{X}$ and X), $L_{reg}$ is the regression loss (huber loss between y and $\hat{y}$) [37], and $L_{pass}$ is the compactness regularizer defined in Section 2.4.3. The coefficients $\alpha \left(t\right)$ and $\beta \left(t\right)$ are dynamically scheduled over training so that the optimization focus gradually shifts from reconstruction to regression. According to the implementation, $\beta \left(t\right)$ decreases progressively, whereas $\beta \left(t\right)$ increases correspondingly.

(10) $$Huber\left(y,\hat{y}\right)=\left\{\begin{array}{c} \frac{1}{2}{\left(y-\hat{y}\right)}^{2},\;\left|y-\hat{y}\right|\leq \delta \\ \delta \left|y-\hat{y}\right|-\frac{1}{2}\delta^{2},\;otherwise \end{array}\right.$$

During training, the relative importance of reconstruction is gradually reduced, whereas the contribution of SSC prediction is increased. In addition, a staged freezing strategy is adopted. In Stage 0, all modules were trained jointly so that the model could form an initial distribution of band importance under the combined influence of reconstruction regularization and the prediction objective. In Stage 1, the attention modules were frozen, while the band-selection, reconstruction, and regression components continued to be optimized, allowing the selected bands to further converge toward the prediction target within a more stable representational space and under reconstruction-assisted regularization. At the end of this stage, the model weights corresponding to the best validation performance were retained, and the selection frequency of each wavelength was counted on the training and validation subsets; the top-K most frequently selected bands were then retained as the final discrete wavelength subset. In Stage 2, all modules except the regression branch were frozen, and the regression model was further fine-tuned using only the fixed top-K wavelengths.

In summary, the proposed progressive stabilization strategy should not be viewed as an additional modeling objective, but rather as the training-stabilization mechanism of the joint framework. Stage 0 explores potentially useful wavelengths, Stage 1 stabilizes the selection process and determines the final wavelength subset under reconstruction-assisted regularization, and Stage 2 performs regression-oriented fine-tuning with fixed bands. Through this staged “exploration–stabilization–refinement” procedure, the framework can transition more reliably from soft selection to discrete deployable wavelengths, thereby improving the consistency between compact-band learning and the final SSC prediction task.

### 2.6. Software and Hardware Environment

All experiments were conducted on a workstation equipped with an NVIDIA GeForce RTX 4090 graphics processing unit (24 GB). The operating system was Ubuntu 22.04.5 LTS. Data preprocessing, model development, and analysis were performed in Python 3.9, using PyTorch 2.5.1 and torchvision 0.20.1. To maintain a consistent experimental platform, relatively unified training settings were used across model groups. The batch size was set to 8 for all models, and the initial learning rate was fixed at 1 × 10^−3^. Reconstruction models (including Rec-only, BS-Net-Conv, etc.) were trained for 60 epochs, regression models (including Reg-only, ResNet18, etc.) for 20 epochs, and the joint model for 80 epochs. This configuration was sufficient for hyperspectral image loading, patch-based training, and subsequent reconstruction and regression optimization.

### 2.7. Quantitative Evaluation Criteria

We evaluated reconstruction quality and SSC prediction accuracy using commonly adopted metrics in hyperspectral imaging and fruit-quality regression studies [38,39,40,41]. For reconstruction, let X and $\hat{X}$ denote the ground-truth and reconstructed cubes, with C bands and spatial size H×W. The mean squared error (MSE) and mean absolute error (MAE) are defined as

(11) $$MSE=\frac{1}{CHW}\sum_{c=1}^{C}\sum_{h,w}{\left({\hat{X}}_{c,h,w}-X_{c,h,w}\right)}^{2}$$

(12) $$MAE=\frac{1}{CHW}\sum_{c=1}^{C}\sum_{h,w}\left|{\hat{X}}_{c,h,w}-X_{c,h,w}\right|$$

Peak signal-to-noise ratio (PSNR) is

(13) $$PSNR=10log10(\frac{{MAE}^{2}}{MSE})$$

where MAX=1 in our normalized reflectance scale. Structural similarity index (SSIM) measures perceptual similarity between two images and is computed here band-wise and averaged over bands [38]:

(14) $$SSIM\left(x,y\right)=\frac{\left(2\mu_{x}\mu_{y}+C_{1}\right)\left(2\sigma_{xy}+C_{2}\right)}{\left(\mu_{x}^{2}+\mu_{y}^{2}+C_{1}\right)\left(\sigma_{x}^{2}+\sigma_{y}^{2}+C_{2}\right)}$$

Spectral correlation coefficient (SCC) evaluates the Pearson correlation between reconstructed and ground-truth spectra at each pixel and then averages over all pixels:

(15) $$SCC=\frac{1}{HW}\sum_{h,w}\frac{\left(x_{h,w}-{\bar{x}}_{h,w}\right)\left({\hat{x}}_{h,w}-{\bar{\hat{x}}}_{h,w}\right)}{{\left\|x_{h,w}-{\bar{x}}_{h,w}\right\|}_{2}{\left\|{\hat{x}}_{h,w}-{\bar{\hat{x}}}_{h,w}\right\|}_{2}}$$

where $x_{h,w}$ $\in R^{\left\{C\right\}}$ and ${\hat{x}}_{h,w}$ ∈ $R^{\left\{C\right\}}$ are spectral vectors at pixel (h,w), and bars denote the mean over the spectral dimension. Spectral angle mapper (SAM) quantifies angular similarity between spectra [39]:

(16) $$SAM=\frac{1}{HW}\sum_{h,w}\left(\frac{x_{h,w}\cdot {\hat{x}}_{h,w}}{{\left\|x_{h,w}\right\|}_{2}{\left\|{\hat{x}}_{h,w}\right\|}_{2}}\right)$$

For SSC prediction, we report MAE, RMSE, coefficient of determination (R^2^), and residual predictive deviation (RPD).

(17) $$RMSE=\sqrt{MSE}$$

(18) $$R^{2}=1-\frac{\sum_{i=1}^{N}{\left(y_{i}-{\hat{y}}_{i}\right)}^{2}}{\sum_{i=1}^{N}{\left(y_{i}-\bar{y}\right)}^{2}}$$

(19) $$RPD=\frac{SD(y)}{RMSE}$$

where $y_{i}$ and ${\hat{y}}_{i}$ are the measured and predicted SSC values for sample i, N is the number of test samples, $\bar{y}$ is the mean measured SSC of the test set, and SD(y) denotes the standard deviation of the measured SSC values in the test set. Lower MAE and RMSE indicate smaller prediction errors, whereas higher R^2^ and RPD indicate better regression performance.

In this study, these prediction metrics quantify the agreement between the SSC values predicted from intact-fruit VNIR hyperspectral images and the destructively measured SSC reference values obtained by refractometry. MAE and RMSE describe the prediction error in °Brix; R^2^ indicates the proportion of variation in the measured SSC reference values explained by the model; and RPD reflects the ratio between the standard deviation of the measured SSC values and the prediction error.

According to commonly used heuristic criteria in agricultural and food-related NIR/HSI studies, external-validation R^2^ values of 0.66–0.81 are generally interpreted as indicating approximate quantitative prediction, values of 0.82–0.90 as good prediction, and values above 0.91 as excellent prediction. For RPD, values below 1.5 are typically considered insufficient for prediction, values of 1.5–2.0 indicate discrimination between high and low values, values of 2.0–2.5 indicate approximate quantitative prediction or rough screening, values of 2.5–3.0 indicate good quantitative prediction, and values above 3.0 indicate excellent prediction [40,41].

## 3. Results

### 3.1. Optimization Behavior During Training

**Figure 3:**
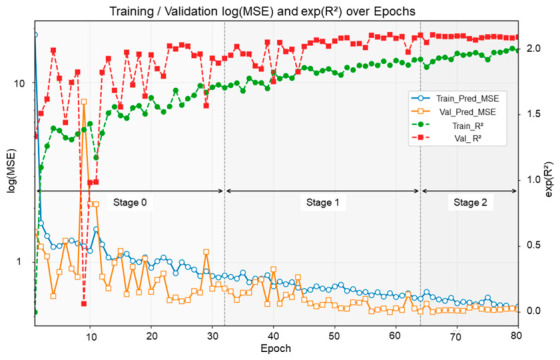
Training dynamics of the proposed framework. The curves show the evolution of training and validation prediction error and R^2^ across epochs. The vertical dashed lines separate the three scheduled stages, illustrating the progressive transition from joint exploration to regression fine-tuning with fixed bands.

The training dynamics of the proposed framework are shown in Figure 3. Overall, both the training and validation curves indicate a stable optimization process under the three-stage schedule. As training progressed, the training prediction error decreased steadily, while the validation prediction error showed an overall downward trend and gradually stabilized at a lower level. In parallel, both training and validation R^2^ increased progressively, indicating a continuous improvement in the model’s ability to explain SSC variance. Although the validation curves fluctuated more than the training curves, which is expected given the smaller size of the validation subset, no pronounced train–validation divergence was observed, suggesting that the staged optimization strategy did not introduce obvious overfitting.

Figure 3 also reveals distinct functional roles for the three training stages. In Stage 0, all modules were optimized jointly, allowing the model to rapidly reduce prediction error while establishing an initial compact representation. In Stage 1, after the attention modules were frozen, the optimization became more stable, with prediction error continuing to decrease and R^2^ continuing to improve, indicating that band selection further converged toward the prediction objective within a more stable representational space. In Stage 2, after the final band subset was fixed, only the regression branch was fine-tuned, and the performance metrics improved more gradually before converging. Taken together, these results suggest that the progressive training strategy effectively supported the transition from early band exploration to SSC-oriented regression refinement with fixed bands.

### 3.2. Compact-Band Operating Point and Spectral Interpretability

After analyzing the optimization behavior during training, we next examined the outcome of compact-band learning from two complementary perspectives: the operating point defined by the number of retained bands, and the spectral distribution of the resulting subset. This analysis is important because the objective of the proposed framework is not simply to minimize the number of wavelengths, but to obtain a compact subset that still preserves sufficient information for both SSC prediction and reconstruction. Therefore, band selection was interpreted here as a trade-off problem among spectral compactness, reconstruction fidelity, and downstream predictive utility.

**Figure 4:**
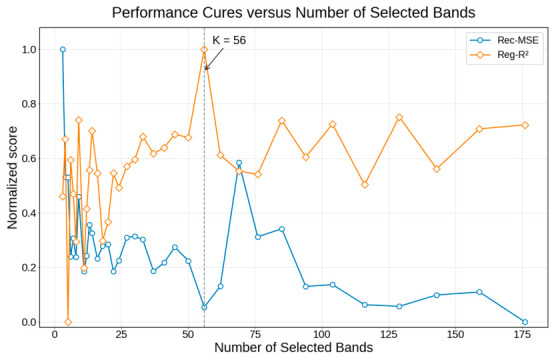
Trade-off between reconstruction error and prediction performance across different numbers of selected bands. The figure shows the normalized reconstruction MSE (Rec-MSE; lower is better) and normalized SSC prediction R^2^ (Reg R^2^; higher is better) as functions of the number of selected bands. The vertical dashed line indicates the adopted operating point at K=56, where the proposed framework achieved a practically favorable balance between reconstruction fidelity and SSC prediction performance.

Figure 4 summarizes how reconstruction-related and prediction-related metrics changed with the number of selected bands. When the band budget was extremely small, the normalized curves fluctuated markedly, indicating that the retained spectral information was insufficient to support stable reconstruction and SSC regression. As the number of selected bands increased, both groups of metrics became progressively more stable and more balanced. In particular, around K=56, the prediction performance had already reached a near-plateau level, whereas the reconstruction metrics had improved substantially relative to more aggressively compressed settings. Beyond this point, further increasing the number of bands brought only limited overall benefit relative to the additional spectral cost. Therefore, K=56 was adopted as the operating point in the subsequent analyses, not because it was the single mathematically optimal value under every metric, but because it provided a practically favorable balance between compactness, reconstruction fidelity, and SSC prediction performance.

**Figure 5:**
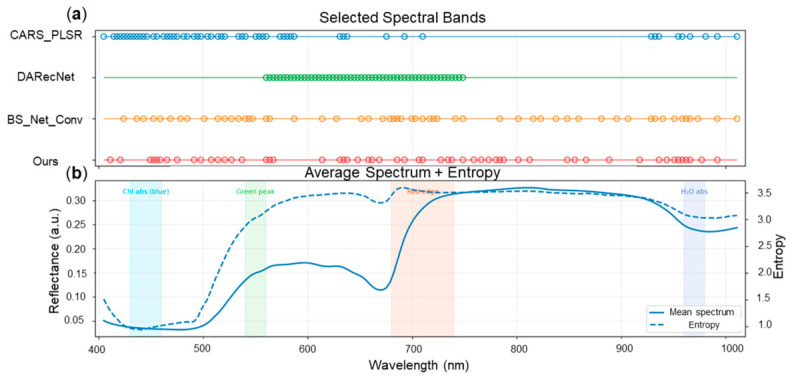
Spectral distribution of selected bands and their relation to the average spectrum and spectral entropy. (**a**) Comparison of the wavelength subsets selected by the proposed method, BS-Net-Conv, DARecNet, and CARS-PLSR. (**b**) Mean reflectance spectrum and spectral entropy of the dataset, with highlighted spectral regions corresponding to representative pigment-, red-edge-, and water-related responses.

The spectral distribution of the selected subsets is shown in Figure 5 and listed in Table 2. Compared with the reference methods, the proposed approach did not concentrate the retained wavelengths within a single narrow interval. Instead, the selected bands were distributed across several spectrally meaningful regions of the VNIR range, including the blue absorption region associated with pigment response, the green reflectance region, the red-edge transition, and the long-wavelength region related to water- and tissue-associated optical variation [20,42,43,44]. This broader coverage suggests that the model did not rely on one localized spectral cue alone, but retained complementary information from multiple parts of the spectrum that may jointly support SSC estimation in intact fruit.

This result is consistent with the task-driven nature of the proposed framework. For intact citrus fruit, SSC is not expected to correspond to a single isolated wavelength feature; rather, it is inferred indirectly through coupled spectral responses related to pigment status, maturity progression, tissue structure, and water-associated absorption behavior. In this context, a compact subset distributed across multiple regions is more plausible than a highly contiguous block if the goal is to preserve predictive utility under strong spectral compression. By contrast, DARecNet tended to concentrate its selected bands in a relatively continuous middle-range interval, whereas CARS-PLSR and BS-Net-Conv showed different degrees of clustering and sparsity. These differences indicate that different selection mechanisms emphasize different criteria, such as statistical screening, reconstruction preference, or task-oriented compact representation learning.

From an interpretability perspective, the selected subset obtained by the proposed method should be understood as a compact and task-relevant spectral design rather than as a definitive set of universal SSC marker wavelengths. Its interpretability lies mainly in two aspects. First, the selected bands show broad yet structured coverage across physically meaningful spectral regions instead of collapsing to a narrow local interval. Second, this distribution is consistent with the reconstruction-assisted, task-driven objective of the framework, which encourages the retained wavelengths to remain informative for both full-band information recovery and SSC prediction. Therefore, the results support the view that the proposed method can provide not only a compact input for regression, but also application-oriented candidate spectral regions for future multispectral citrus-quality sensing.

### 3.3. Reconstruction Quality in Spatial and Spectral Domains

To evaluate whether the compact band subset retained sufficient information beyond direct SSC regression, reconstruction quality was examined from both spatial and spectral perspectives. Here, reconstruction refers to recovering the full C-band hyperspectral cube from a reduced or complete input representation. For clarity, K→C denotes reconstructing the original C-band cube from K selected bands; this is the compact-input setting and the main scenario relevant to practical multispectral deployment. In contrast, C→C denotes reconstructing the C-band cube from the full C-band input; this setting is less constrained and is included mainly as a reference for reconstruction capability rather than as a fully matched comparison to compact-input reconstruction.

**Figure 6:**
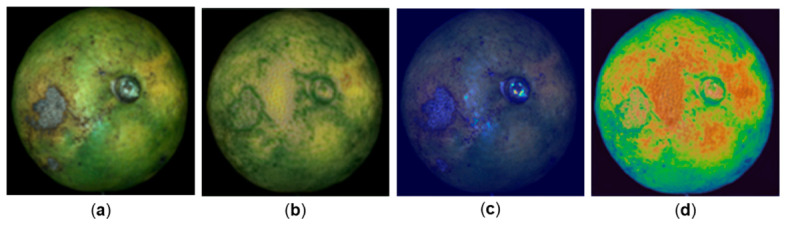
Representative spatial-domain reconstruction results of the proposed framework. (**a**) Original fruit image before reconstruction. (**b**) Reconstructed image generated from the selected-band input. (**c**) Reconstruction-loss heatmap, showing the spatial distribution of reconstruction error, where relatively warmer/brighter colors indicate larger local reconstruction discrepancies and cooler/darker colors indicate smaller errors. (**d**) Attention heatmap, showing the regions emphasized by the model during compact-band learning and reconstruction, where warmer colors denote higher attention intensity and cooler colors denote lower attention intensity.

Figure 6 presents representative spatial-domain results under the compact-input reconstruction setting. The reconstructed image preserves the major fruit contour, broad texture distribution, and overall intensity pattern of the original sample. Although some local fine details are inevitably smoothed, the main spatial structure remains well recovered. The reconstruction-error heatmap further shows that the error is not uniformly distributed across the fruit surface, but is concentrated in relatively complex local regions. The attention heatmap indicates that the network places greater emphasis on spatially informative areas during compact-band learning and reconstruction. Taken together, these visual examples suggest that the proposed framework does not merely generate a blurred approximation of the fruit image, but restores spatially meaningful structure from the compact spectral input.

**Figure 7:**
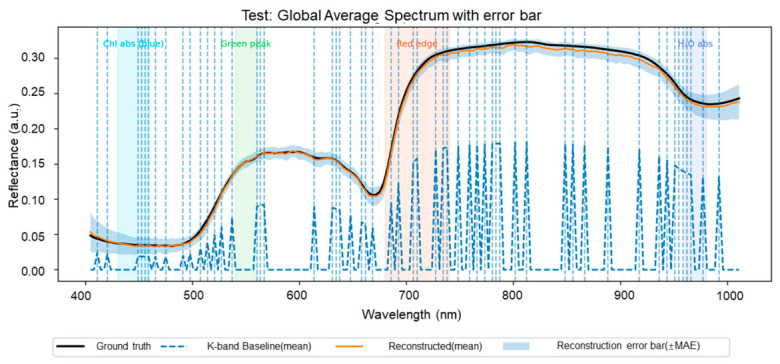
Spectral-domain reconstruction on the test set under the compact-input setting (K→C). The figure compares the ground-truth mean spectrum, the directly observed K-band baseline, and the reconstructed mean spectrum. The shaded band indicates the reconstruction-error range, and the vertical dashed lines mark the selected wavelengths, illustrating how the proposed framework restores missing spectral information beyond the retained bands.

Figure 7 further evaluates reconstruction quality in the spectral domain. On the test set, the reconstructed mean spectrum closely follows the ground-truth spectrum across the visible region, the red-edge transition, the near-infrared plateau, and the water-related absorption decline at longer wavelengths. The shaded error band remains relatively narrow over most wavelengths, indicating that the reconstruction branch preserves not only the average spectral shape, but also a reasonable degree of across-sample variation. Compared with the K-band baseline, which contains only the directly observed selected wavelengths, the reconstructed spectrum restores substantial missing information between retained bands. This result is important because it shows that the model does not simply pass through the observed wavelengths, but learns to recover a fuller spectral representation from the compact input.

The quantitative results in Table 3 provide a clearer view of this trade-off. Under the more challenging K→C setting, the proposed joint model achieved strong reconstruction performance (MAE = 0.011, MSE = 0.00023, PSNR = 36.47, and SSIM = 0.89) and substantially outperformed the K-band baseline, confirming that the selected 56-band input retained enough information to support high-fidelity full-band recovery. The Rec-only (K→C) model achieved the best MAE, MSE, and PSNR among the compared methods, which is expected because it was optimized solely for reconstruction. By contrast, the proposed joint framework was designed to balance reconstruction with downstream SSC prediction, and therefore accepts a limited loss in pure reconstruction optimality in exchange for a more task-relevant compact representation.

The comparison with the C→C reference methods should be interpreted carefully. Models such as BS-Net-Conv and DARecNet reconstruct from the full-band input, whereas the proposed framework reconstructs the full cube using only K selected bands. Therefore, the C→C setting has an inherent information advantage, especially for metrics that strongly reflect global structural consistency or spectral-shape similarity. Even under this more constrained compact-input setting, however, the proposed model remained competitive and achieved markedly better spectral recovery than the direct K-band observation. Overall, the reconstruction results support the reconstruction-assisted design of the framework: the learned compact subset is not only useful for SSC prediction, but also sufficiently informative to recover a large portion of the original hyperspectral structure.

### 3.4. SSC Prediction Accuracy and Model Comparison

**Figure 8:**
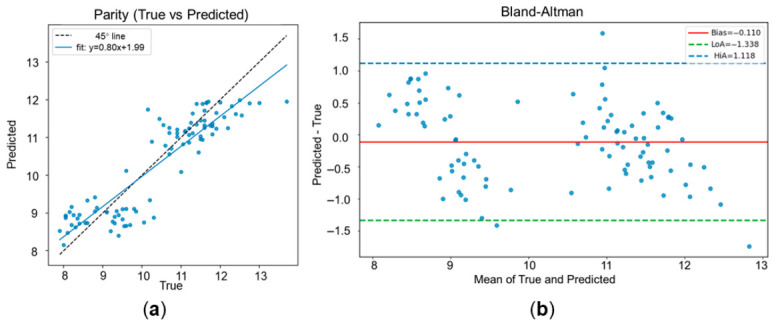
SSC prediction performance on the test set. (**a**) Parity plot comparing the SSC values predicted by the proposed VNIR-HSI model with the SSC values measured destructively by the refractometric method. The horizontal axis represents the measured SSC reference values obtained from extracted juice, whereas the vertical axis represents the model-predicted SSC values from intact-fruit hyperspectral images. Each point corresponds to one fruit sample in the independent test set, and the 1:1 line indicates perfect agreement between prediction and reference measurement. (**b**) Bland–Altman plot showing the agreement between predicted and measured SSC values. The horizontal axis represents the mean of the predicted and measured SSC values for each sample, whereas the vertical axis represents the prediction error, calculated as predicted SSC minus measured SSC. The solid horizontal line indicates the mean prediction bias, and the dashed lines indicate the 95% limits of agreement.

After examining whether the selected compact bands could preserve sufficient information for full-band reconstruction, we next evaluated their downstream utility for SSC prediction. Figure 8 compares the non-destructive SSC predictions with the SSC values obtained from juice analysis by the refractometric method. In Figure 8a, each point represents one fruit sample in the independent test set. The horizontal axis corresponds to the SSC measured destructively from extracted juice, whereas the vertical axis corresponds to the SSC predicted from the VNIR hyperspectral image of the intact fruit. Therefore, the parity plot directly evaluates whether the non-destructive prediction agrees with the physicochemical reference measurement. The predicted SSC values generally followed the measured values across the examined range, indicating that the model captured the overall SSC variation in the fruit samples. Figure 8b further evaluates the agreement between prediction and reference measurement using Bland–Altman analysis. The residual was calculated as predicted SSC minus measured SSC and plotted against the mean of the predicted and measured values. This plot is useful for evaluating whether the model shows systematic bias across the SSC range. The residuals were centered close to zero, with no obvious systematic trend across the examined SSC range. Although some dispersion remained at the two ends of the distribution, the overall pattern suggests moderate agreement between VNIR-HSI prediction and refractometric SSC measurement under the present setting.

The quantitative results are summarized in Table 4. The proposed framework achieved a test MAE of 0.52 °Brix, an RMSE of 0.63 °Brix, an R^2^ of 0.80, and an RPD of 2.26. Under commonly used heuristic interpretations in agricultural and food-related NIR studies, such a result is more reasonably viewed as approximate quantitative prediction or rough screening rather than excellent prediction [40,41]. Therefore, the present result should not be described as having deployment-ready accuracy for arbitrary unknown samples. At the same time, it is more appropriately interpreted as a competitive result obtained under substantial spectral compression on the present held-out test set, rather than as a clearly unsuitable outcome.

More importantly, this level of predictive performance was achieved using only 56 selected bands from the original 176-band hyperspectral input. From the perspective of this study, the main value of the proposed framework is therefore not merely that it produced the lowest error among the compared models, but that it maintained prediction performance close to the full-band baseline under substantial spectral compression. This observation is consistent with the reconstruction results in Section 3.4 and supports the central premise of the framework: the selected compact subset remains sufficiently informative for downstream SSC estimation while reducing spectral dimensionality considerably. Consequently, the proposed method offers a food-quality-motivated proof of concept for compact citrus sensing and future multispectral system design.

## 4. Discussion

### 4.1. Why Joint Learning Helps Under Band-Limited Settings

The results in Table 5 help explain why the proposed framework remains effective when the available band budget is strongly constrained. The purpose of this comparison is not simply to show that one model yields the lowest numerical error, but to clarify how different band-selection strategies affect downstream SSC prediction under the same compact-input setting. In particular, the table compares the proposed joint framework with a full-band regression baseline, a direct regression model using a fixed compact subset, and several alternative band-selection strategies combined with the same regression head.

A first important observation is that the full-band regression baseline remained highly competitive. Reg-only (full bands) achieved performance very close to that of the proposed model, indicating that the original 176-band input already contains sufficient task-relevant information for accurate SSC estimation. Therefore, the main value of the proposed framework should not be interpreted as dramatically outperforming an unconstrained full-band model. Rather, its significance lies in achieving a nearly comparable prediction level using only 56 selected bands, which supports the feasibility of substantial spectral compression without marked loss of predictive utility.

A second key observation is that direct regression on a compressed subset was less effective than the proposed joint-learning strategy. Compared with Reg-only (K bands), which used the fixed Top-K subset derived from band-selection frequencies, the proposed framework achieved lower prediction error and better goodness-of-fit. This result suggests that simply reducing the input to a smaller subset is not sufficient by itself. When compression is performed without reconstruction-assisted learning, part of the information useful for SSC estimation is more likely to be lost. In contrast, the reconstruction branch acts as an auxiliary constraint that discourages the selector from collapsing toward an overly narrow or unstable subset, while the regression objective keeps the retained wavelengths aligned with the downstream SSC task. In this sense, the proposed framework learns a compact representation that is not only sparse, but also informative and task-relevant.

The comparison with BS + Reg-only, DARecNet + Reg-only, CARS-PLSR + Reg-only, and Random band + Reg-only further highlights the importance of task-driven band learning. Weight-based Top-K, entropy-based Top-K, heuristic statistical selection, and random selection can all produce reduced inputs, but they do not optimize compact-band formation and SSC prediction in a unified manner. As a result, the resulting subsets may retain bands that are useful for generic compression, reconstruction preference, or statistical screening, but are not necessarily the most effective combination for downstream SSC regression. The stronger performance of the proposed model therefore supports the view that band selection should be guided as directly as possible by the final prediction objective while still being regularized to preserve broader spectral information.

Overall, the ablation results suggest that the advantage of joint learning in this study does not arise from compression alone, but from how compression is learned. Under a fixed band budget, a compact subset becomes more useful when it is shaped jointly by reconstruction-assisted information preservation and SSC-oriented supervision. This is precisely the role of the proposed framework: not merely to select fewer wavelengths, but to learn a compact spectral representation that remains informative for non-destructive citrus quality evaluation under band-limited sensing conditions.

### 4.2. Interpretability of the Selected Bands

Interpretability is an important consideration in compact-band learning, especially for food-quality applications, where the selected wavelengths are often expected to retain at least partial physical meaning [2,3,4,14,17]. In the present study, the interpretability of the learned compact subset should be understood from two complementary perspectives: selection stability and spectral relevance. The former addresses whether the retained bands emerge consistently under the training objective rather than by chance, whereas the latter concerns whether the selected wavelengths are distributed in a manner that is plausible for intact-fruit SSC estimation.

**Figure 9:**
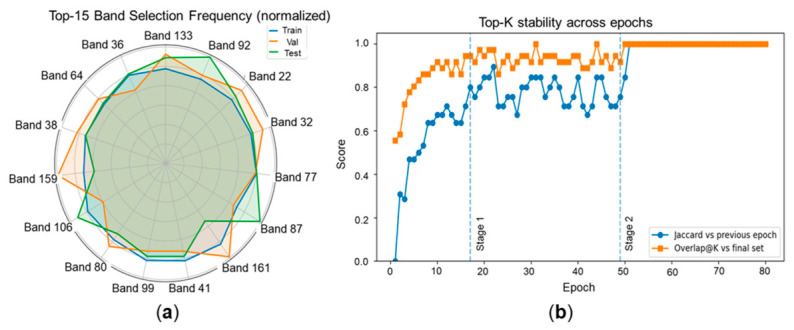
Stability analysis of the learned compact-band subset. (**a**) Normalized top-15 band-selection frequencies in the training, validation, and test splits, showing that the most frequently retained wavelengths were broadly consistent across data partitions. (**b**) Stability of the Top-K subset across training epochs, measured by the Jaccard similarity to the previous epoch and the overlap at K with the final selected subset. The curves illustrate the progression from early exploration to stable convergence and finally to a fixed compact-band configuration.

Figure 9 first provides evidence from the data-driven perspective. The normalized top-15 band-selection frequencies were broadly consistent across the training, validation, and test splits, indicating that the most frequently retained wavelengths were not dominated by a single subset. In addition, the stability curves show that band selection evolved from an exploratory stage to a more stable stage and finally converged to a fixed Top-K subset. Together, these results suggest that the learned compact-band configuration was not an artifact of one optimization step or one specific split, but the result of repeated preference for similar spectral regions under the joint reconstruction-and-prediction objective.

From the spectral perspective, the interpretability of the selected subset should be considered together with the distribution analysis in Section 3.2. The retained wavelengths were not collapsed into one narrow interval; instead, they spanned several spectrally meaningful regions across the visible, red-edge, and near-infrared ranges. This pattern is plausible for intact citrus SSC sensing. SSC is not expected to be represented by a single isolated VNIR absorption feature; rather, it is inferred indirectly through coupled spectral responses associated with pigment status, maturity progression, tissue structure, and water-related absorption behavior [11,12,13,20,42,43,44]. Therefore, a compact subset distributed across multiple complementary spectral regions is more consistent with the nature of intact-fruit SSC estimation than a highly localized wavelength block.

At the same time, this interpretability should not be overstated. The selected bands should not be viewed as universally optimal SSC wavelengths for all citrus fruit or all acquisition settings. Instead, they are better interpreted as task-relevant wavelengths learned under the present data distribution, imaging configuration, and optimization strategy. Their interpretability lies mainly in two aspects: first, the subset is stable enough to suggest non-random preference under training; second, its spectral coverage is sufficiently broad and structured to remain compatible with both full-band reconstruction and downstream SSC prediction. In this sense, the proposed framework provides not only a compact input representation, but also food-quality-related candidate spectral regions for future compact citrus sensing.

### 4.3. Implications for Postharvest Quality Control and Compact Sensor Design

An important practical implication of the present study is that informative SSC prediction did not require retaining the full 176-band hyperspectral input once a task-relevant compact subset had been learned. In the present dataset, the proposed joint framework maintained prediction performance close to the full-band regression baseline while operating on only 56 selected bands. From a food-sector perspective, this result is relevant to postharvest grading, rapid screening, and precision supply, where compact optical sensing is preferable to full laboratory hyperspectral acquisition [45].

As an exploratory analysis beyond the main operating point, we additionally examined a more aggressive setting with only 25 retained bands. Under this condition, the model still achieved useful SSC prediction performance on the test set (MAE = 0.51 °Brix, R^2^ = 0.81), while reconstruction fidelity showed a moderate decline relative to the adopted K=56 setting (PSNR = 35.37 dB; SSIM = 0.87). This exploratory result suggests that further spectral simplification may still be feasible when prediction is prioritized over full-spectrum recovery, but it also highlights a trade-off between sensing simplicity and retained spectral information.

These implications should nevertheless be interpreted with appropriate caution. The present work does not yet demonstrate a deployable multispectral device, nor does it show that the same selected subset would remain optimal across different cultivars, batches, instruments, or field conditions. Rather, the current results indicate that the proposed framework can function as a data-driven band-screening strategy for identifying candidate spectral regions that warrant further validation in food-quality control applications [45].

From an engineering perspective, the spectral distribution of the selected bands is also informative. Because the retained wavelengths were spread across multiple complementary spectral regions rather than concentrated within one narrow interval, a future multispectral design should ideally preserve this diversity instead of collapsing to one local window. In practical terms, this may help guide the choice of optical filters, illumination wavelengths, or sensor response windows in prototype systems intended for citrus quality sorting.

## 5. Conclusions

This study developed a reconstruction-assisted, attention-guided band-selection framework for non-destructive SSC prediction in Shimen honey mandarins using VNIR hyperspectral imaging. Under the present dataset and acquisition setting, the proposed method selected 56 bands from the original 176-band input and maintained a prediction performance close to the full-band baseline while also supporting the reconstruction of the full-band cube from the compact input. These results suggest that substantial spectral compression can be achieved with limited loss of predictive performance under the current VNIR setting.

The present work should be interpreted as a food-quality-motivated methodological proof of concept rather than as a deployment-ready industrial solution. Its main contribution lies in showing that compact-band learning, jointly constrained by reconstruction and prediction, can retain much of the predictive utility of full-band hyperspectral data for non-destructive citrus quality evaluation. Future work should test the robustness of the selected spectral regions across broader datasets, controlled sampling conditions, additional quality traits, and spectral ranges better suited to acidity-related prediction.

## Figures and Tables

**Table 1:** Summary statistics of SSC labels and hyperspectral spectra for the training, validation, and test subsets. The table reports the sample number, label range, mean, and standard deviation of SSC, as well as the mean spectral intensity, spectral standard deviation, and the L2 distance to the global spectral distribution for each subset.

Dataset	N	SSC Label				Hyperspectral Spectra		
		Min	Max	Mean	Std	Mean	Std	L2 to Global
Train	765	6.80	14.10	10.23	1.35	0.20	0.13	0.014
Val	93	7.60	14.00	10.54	1.45	0.20	0.12	0.121
Test	94	7.90	13.70	10.39	1.44	0.20	0.12	0.049

**Table 2:** Selected band indices (K=56) for different band-selection methods. Comparison of the compact wavelength subsets obtained by the proposed method, BS-Net-Conv, DARecNet, and CARS-PLSR.

Model	Selected Band Index
Ours	[2, 5, 14, 15, 16, 17, 19, 22, 27, 29, 32, 34, 36, 38, 41, 48, 49, 50, 64, 69, 70, 71, 74, 77, 78, 80, 85, 87, 91, 92, 97, 99, 100, 103, 106, 108, 110, 112, 113, 114, 118, 121, 131, 133, 136, 142, 150, 155, 157, 159, 160, 161, 162, 163, 166, 170]
BS-Net-Conv	[6, 10, 12, 15, 17, 20, 23, 25, 30, 34, 36, 38, 40, 42, 43, 44, 48, 49, 56, 64, 68, 75, 77, 81, 83, 84, 85, 86, 89, 90, 92, 94, 95, 96, 101, 103, 113, 118, 122, 124, 128, 131, 134, 140, 144, 147, 153, 154, 156, 159, 161, 162, 163, 165, 170, 175]
DARecNet	[48, 49, 50, 51, 52, 53, 54, 55, 56, 57, 58, 59, 60, 61, 62, 63, 64, 65, 66, 67, 68, 69, 70, 71, 72, 73, 74, 75, 76, 77, 78, 79, 80, 81, 82, 83, 84, 85, 86, 87, 88, 89, 90, 91, 92, 93, 94, 95, 96, 97, 98, 99, 100, 101, 102, 103]
CARS-PLSR	[0, 3, 4, 5, 6, 7, 8, 9, 10, 11, 12, 13, 15, 16, 18, 19, 20, 21, 22, 24, 25, 27, 28, 29, 31, 32, 34, 35, 36, 40, 41, 42, 45, 46, 47, 48, 52, 53, 54, 55, 56, 69, 70, 71, 82, 87, 92, 153, 154, 155, 160, 161, 163, 167, 170, 175]

**Table 3:** Reconstruction performance on the test set under compact-band (K→C) and full-input (C→C) settings. Bold values indicate the best performance.

Model	MAE	MSE	PSNR	SSIM	SCC	SAM
Ours (K→C)	0.011	0.00023	36.47	0.89	0.71	7.71
K-band baseline	0.165	0.06518	12.03	0.29	0.24	56.07
Rec-only (K→C)	**0.009**	**0.00019**	**37.59**	0.91	0.73	6.87
BS-Net-Conv (C→C)	0.020	0.00069	31.64	0.86	0.99	2.50
DARecNet (C→C)	0.013	0.00027	35.75	**0.98**	**0.99**	**1.45**

**Table 4:** SSC prediction performance on the test set for the proposed framework and the compared baselines. Bold values indicate the best performance.

Model	MAE	RMSE	R^2^	RPD
Ours	**0.52**	**0.63**	**0.80**	**2.26**
Random Forest	0.64	0.79	0.69	1.81
CARS-PLSR	0.53	0.66	0.79	2.19
MLP	0.66	0.80	0.69	1.79
ResNet18	0.55	0.67	0.78	2.14
ViT	0.67	0.79	0.69	1.82

**Table 5:** Ablation of band-learning strategies for SSC prediction under a fixed compact-band budget. The table compares the proposed joint framework with a full-band regression baseline, a direct regression model using a fixed Top-K subset, and several alternative band-selection strategies combined with the same regression head. Bold values indicate the best performance.

Model	Band Selection	MAE	RMSE	R^2^	RPD
Ours	Task-driven	0.52	**0.633**	**0.804**	2.26
Reg-only (full bands)	None	**0.51**	0.634	0.803	**2.27**
Reg-only (K bands)	Frequency-based Top-K	0.54	0.659	0.788	2.18
BS + Reg-only	Weight-based Top-K	0.53	0.644	0.797	2.23
DARecNet + Reg-only	Entropy-based Top-K	0.55	0.687	0.769	2.09
CARS-PLSR + Reg-only	Heuristic Statistical	0.60	0.744	0.730	1.93
Random band + Reg-only	Random	0.62	0.771	0.710	1.87

## Data Availability

The original contributions presented in the study are included in the article. Further inquiries can be directed to the corresponding authors.
